# Membrane Proteins: Function, Structure, and Dynamics

**DOI:** 10.3390/membranes13120904

**Published:** 2023-12-09

**Authors:** Yosuke Senju, Shiro Suetsugu

**Affiliations:** 1 Research Institute for Interdisciplinary Science (RIIS), Okayama University, Okayama 700-8530, Japan; 2 Division of Biological Science, Graduate School of Science and Technology, Nara Institute of Science and Technology, Nara 630-0192, Japan; 3 Data Science Center, Nara Institute of Science and Technology, Nara 630-0192, Japan; 4 Center for Digital Green-innovation, Nara Institute of Science and Technology, Nara 630-0192, Japan

Plasma and intracellular membranes are characterized by different lipid compositions that enable proteins to localize to distinct subcellular compartments [1]. Many proteins interact with cellular and subcellular membranes. These include transmembrane proteins (TMEMs) (e.g., ion channels, transmembrane receptors, and transporters), which integrate into lipid bilayers to transport molecules and ions across membranes, and peripheral membrane proteins, which associate with membranes via electrostatic and/or hydrophobic interactions by penetrating into lipid bilayers. These lipid–protein interactions determine protein conformations and protein–protein interactions, which in turn precisely regulate the localization and activation of molecular complexes at the respective membranes. These signaling pathways are crucial for various cellular processes, such as membrane trafficking and signal transduction. Membrane proteins have also been implicated in many diseases, such as cancer and Alzheimer’s disease, and can be targeted for drug design; however, given the complexity of the abundant lipid–protein/protein–protein interactions at membranes, the exact molecular and cellular mechanisms underlying the structure and function of membrane proteins remain unclear.

This Special Issue of *Membranes*, entitled “Membrane Proteins: Function, Structure, and Dynamics”, discusses the recent progress in the studies of membrane proteins from various perspectives, including cell biology, biochemistry, biophysics, structural biology, and molecular dynamics (MD) simulations. These studies elucidate the structural and physiological functions of membrane proteins, providing new insights into their fundamental principles. A summary of these studies is as follows.

Kim et al. [2] studied the lipid-scrambling and ion-transport activities of human TMEM16C isoforms. They found that, among the isoforms 1–3, isoforms 1 and 3 transported phosphatidylserine (PS) to the outer leaflet of the membrane, whereas isoform 2 did not. They also found that these results were due to the differences in the surface expression levels of each isoform. The surface expression of isoform 2, which did not exhibit scrambling activity, was approximately five times lower than that of the other two isoforms. Unlike other TMEM16 proteins, TMEM16C isoforms did not show ion channel activity in flux assays or electrophysiological recordings. Thus, they concluded that the N-terminus of TMEM16C isoforms 1 and 3 determines whether they can translocate to the plasma membrane and facilitate the scrambling activity of transporting PS to the outer leaflet.

Li et al. [3] focused on the dimerization of TMEMs in cancer immunotherapy. They described the structures and functions of several TMEMs related to tumor immunity and analyzed the binding properties and functions of these immune checkpoint proteins and their receptors. They also discussed the regulation of TMEM dimerization and its potential as a target for cancer immunotherapy. They believe that understanding the mechanisms underlying TMEM dimerization will provide valuable insights into the development of novel antitumor drugs.

Anosov et al. [4] studied the influence of pH and cytochrome c (cyt c) on the electrical properties of asolectin bilayer lipid membranes. They found that acid phospholipids in asolectin membranes can bind to cyt c. They measured differences in cyt c-induced surface potential and found that as the pH decreased, the adsorption of cyt c molecules on the surface of asolectin membranes increased although no increase in membrane conductance. In contrast, the membrane conductance increased as the pH increased, indicating that these two variables were directly proportional.

Rodrigues et al. [5] aimed to provide an in-depth characterization of the proteins in the outer membrane of uropathogenic *Escherichia coli* (UPEC), with a focus on outer membrane proteins (OMPs) and their role in antimicrobial resistance. They discussed several OMPs were related to antimicrobial resistance. Fluoroquinolones and β-lactams were the antibiotics most affected by OMP-conferred antimicrobial resistance. The authors emphasized that the implementation of new strategies for administering antimicrobial agents, the development of improved antimicrobials, protective vaccines, and specific inhibitors of virulence and resistance factors are crucial in veterinary and human medicine to address UPEC resistance to antimicrobial agents.

Dengler et al. [6] investigated the molecular basis for the cholecystokinin (CCK) action of type 1 CCK receptor (CCK1R) positive allosteric modulator (PAM) ligands. Their findings expanded our understanding of the structure–activity relationships of molecules with tetracyclic scaffolds. They explored structural modifications present in 65 commercially available analogs and conducted an analog-by-catalog expansion of the scaffold. They eliminated the off-target effect observed in this scaffold while maintaining its activity as a PAM of CCK1R in normal and high-cholesterol membrane environments.

Isu et al. [7] reviewed the binding sites of cholesterol within the structures of class C G-protein-coupled receptors (GPCRs) and found that cholesterol is typically bound between the transmembrane dimers of GPCRs and within the surrounding grooves of their transmembrane helices.

Bykhovskaia [8] discussed MD simulations to investigate the pre-fusion protein–lipid complex. The author discussed the dynamics of the SNARE complex between lipid bilayers, interactions of synaptotagmin-1 with lipids and SNARE proteins, and the regulation of complexin in the SNARE complex assembly that controls synaptic vesicle fusion.

Westra et al. [9] performed confinement analyses on simulated random walks and trajectories that exhibited transient confined behavior by optimizing the parameters for various experimental conditions. The authors also developed a tool to visualize confinement areas in heat maps that allowed the spatial mapping of confinement hotspots relative to subcellular markers. Using these optimized tools, they reliably detected the subdiffusive behavior of different membrane components and revealed the different confinement properties of the two types of glutamate receptors in neurons. Their results provide a systematic analysis of the influence of different parameters used for detecting temporal confinement in single-molecule trajectories, and a visualization tool for mapping confinement zones in the cellular context.

Selikhanov et al. [10] proposed a rational design approach for genetically engineering the photosynthetic reaction center in *Cereibacter sphaeroides* by introducing disulfide bonds between its α-helices. This modification increased the thermal stability and functional activity of some mutants of photosynthetic pigment–protein complexes, while effectively maintaining the photochemical charge separation ability of the genetically modified reaction centers.

Radka [11] discussed the biochemical and biophysical advances in understanding how interfacial enzymes involved in exogenous fatty acid (eFA) metabolism interact with the membrane. The author aimed to provide a molecular mechanistic understanding of how peripheral membrane proteins use conformational changes to precisely regulate their activation, localization, and integration into the membrane, and how these protein–lipid interactions contribute to enzyme catalysis.

Beltran et al. [12] revealed the functional properties shared between borate transporter Bor1p in *Saccharomyces cerevisiae* and SLC4, including lipid-promoted dimerization, sensitivity to stilbene disulfonate-derived inhibitors, the requirements of an acidic residue at the solute binding site, and the conservation of disease-causing mutations.

Jasni et al. [13] aimed to identify and understand the protein structure, function, and interaction of the biological membrane of *Entamoeba histolytica* upon the immune response, which could contribute to vaccine development. Furthermore, they reviewed the current development of vaccines targeting amoebiasis.

Collectively, the studies included in this Special Issue update our current knowledge on the function, structure, and dynamics of membrane proteins. The technical approaches presented here, such as single-particle tracking, structural biology, and MD simulations, will help us understand the spatiotemporal dynamics and physical properties of membrane proteins. These studies provide new insights into the fundamental principles underlying the physiological functions of membrane proteins, such as GPCRs in cells and tissues, and could lead to the development of new drug designs.
